# Description of Pseudomonas stagnisoli sp. nov. and Pseudomonas cimarronensis sp. nov., isolated from freshwater sediments

**DOI:** 10.1099/ijsem.0.007075

**Published:** 2026-02-16

**Authors:** Md. Mehedi Hasan, Noha H. Youssef, Mostafa S. Elshahed, Samuel L. Miller

**Affiliations:** 1 Department of Microbiology and Molecular Genetics, Oklahoma State University, Stillwater, OK, USA

**Keywords:** polyphasic taxonomy, *Pseudomonas*, *Pseudomonas cimarronensis*, *Pseudomonas stagnisoli*, sediment, whole-genome sequencing

## Abstract

A polyphasic taxonomic approach was employed to characterize two novel strains, P3C3^T^ and MAC6^T^, isolated from sediments of the Innovation Way Drive Pond (Stillwater, OK) and the Cimarron River (Perkins, OK), respectively. Both strains were strictly aerobic, Gram-stain-negative, rod-shaped, motile and non-spore-forming bacteria. Strain P3C3^T^ grew optimally at 35 °C, pH 6.0 and 0.5% (v/w) NaCl, while strain MAC6^T^ grew optimally at 30 °C, pH 6.0 and 4.0% (v/w) NaCl. Based on 16S rRNA gene sequence similarity, P3C3^T^ was most closely related to *Pseudomonas alcaligenes* (98.3%), whereas MAC6^T^ was most closely related to *Pseudomonas anguilliseptica* (98.6%). Phylogenetic analysis using the 16S rRNA gene and whole-genome phylogenomic analysis indicated that P3C3^T^ and MAC6^T^ formed distinct branches within the *Pseudomonas* genus. Additionally, overall genomic-based relatedness indices demonstrated that P3C3^T^ and MAC6^T^ and the type strains of close relatives shared average nucleotide identity and digital DNA–DNA hybridization values <95% and <70%, respectively, which are below the currently accepted thresholds for species-level delineation. The genomes of strains P3C3^T^ and MAC6^T^ were 4.3 and 4.2 Mbp, respectively, with G+C contents of 64.5 and 60.5 mol%. Based on polyphasic taxonomic criteria, P3C3^T^ and MAC6^T^ represent two novel species within the genus *Pseudomonas*, for which the names *Pseudomonas stagnisoli* and *Pseudomonas cimarronensis* sp. nov. are proposed. The type strains are P3C3^T^ (=CCM 9468^T^=CECT 31230^T^=DSM 119661^T^) and MAC6^T^ (=CCM 9470^T^=CCUG 78313^T^=CECT 31231^T^), respectively.

## Data Accessibility

The GenBank/EMBL/DDBJ accession numbers for the 16S rRNA gene sequences of strains P3C3^T^ and MAC6^T^ are PV166458 and PV166459, respectively. The GenBank/EMBL/DDBJ accession numbers for the whole-genome sequences of strains P3C3^T^ and MAC6^T^ are GCA_048595745.1 and GCA_049331905.1, respectively. The type strain P3C3^T^ has been deposited in the Czech Collection of Microorganisms (accession number: CCM 9468^T^), the Spanish Type Culture Collection (accession number: CECT 31230^T^) and the German Collection of Microorganisms and Cell Cultures GmbH (accession number: DSMZ 119661^T^). The type strain MAC6^T^ has been deposited in the Czech Collection of Microorganisms (accession number: CCM 9470^T^), the Culture Collection University of Gothenburg (accession number: CCUG 78313^T^) and the Spanish Type Culture Collection (accession number: CECT 31231^T^).

## Introduction

The genus *Pseudomonas* was proposed in 1894 [1] and is currently assigned to the family *Pseudomonadacea*e, order *Pseudomonadales*, class *Gammaproteobacteria* and phylum *Pseudomonadota. Pseudomonas* species are Gram-stain-negative, rod-shaped, non-spore-forming, motile and primarily aerobic [2]. According to the List of Prokaryotic Names with Standing in Nomenclature [3], the genus encompasses 355 validly published species (as of September 2025). Members of the genus *Pseudomonas* have been described from a wide range of aquatic [4,5] and terrestrial [6] habitats. Additionally, *Pseudomonas* species have been isolated from plants [7], insects [8] and animals [9]. Some species are known plant pathogens [10], and others were reported to cause opportunistic infection in humans [11]. The majority of species, however, are free-living, and members of *Pseudomonas* are recognized for their crucial contributions to agricultural and environmental sustainability [12,15]. This study reports the isolation and characterization of two novel isolates (P3C3^T^ and MAC6^T^) from sediments obtained from freshwater environments: the Innovation Way Drive Pond (Stillwater, OK) and the Cimarron River (Perkins, OK). Based on a polyphasic approach, these two isolates represent novel *Pseudomonas* species, for which the names *Pseudomonas stagnisoli* sp. nov. and *Pseudomonas cimarronensis* sp. nov. are herein proposed.

## Isolation and ecology

Strain P3C3^T^ was isolated from a sediment sample collected from a shallow freshwater pond located in Stillwater, OK (36.12° N 97.07° W), and strain MAC6^T^ was isolated from a sediment sample collected from the Cimarron River in Perkins, OK (35.97° N 97.03° W). Sediment samples were serially diluted in PBS (1×) and subsequently spread onto Reasoner’s 2A Agar (R2A), Tryptic Soy Agar (TSA) and MacConkey Agar (MAC) and incubated aerobically at 25 and 30 °C. Following incubation, individual colonies with differing morphological profiles were further sub-cultured until pure isolates were obtained, as evidenced by phase-contrast microscopy (BX51, Olympus, Center Valley, PA). Strain P3C3^T^ was isolated on R2A, whereas strain MAC6^T^ was detected on MAC. After primary isolation, P3C3^T^ and MAC6^T^ were maintained under aerobic conditions on TSA at 35 °C and 30 °C, respectively. Isolates P3C3^T^ and MAC6^T^ were preserved in Microbank cryogenic beads (Prolab Diagnostics, Georgetown, TX) according to the manufacturer’s instructions and stored at −80 °C.

## 16S rRNA gene analysis and phylogeny

Near-full-length 16S rRNA gene sequences were amplified using the universal primers 27F (5′-AGAGTTTGATCCTGGCTCAG-3′) and 1492R (5′-TACGGYTACCTTGTTACGACTT-3′) [16] and Sanger-sequenced at the Oklahoma State University Core Sequencing Facility (Stillwater, OK). These sequences were queried against the EzBioCloud database [17] with blast [18] to identify taxa with the highest percentage sequence similarity to each strain. 16S rRNA gene sequences of P3C3^T^ and MAC6^T^, as well as their close type species relatives that are validly described (with standing in nomenclature) (downloaded from EzBioCloud), were subsequently aligned using muscle [19]. A phylogenetic tree using the maximum-likelihood method [20] with the Tamura–Nei substitution model [21] was constructed with 1,000 bootstrap replications. The 16S rRNA gene phylogenetic tree was visualized using mega 12 [22]. *Entomomonas moraniae* QZS01^T^ (MG768960) was used as an outgroup.

**Fig. 1.**
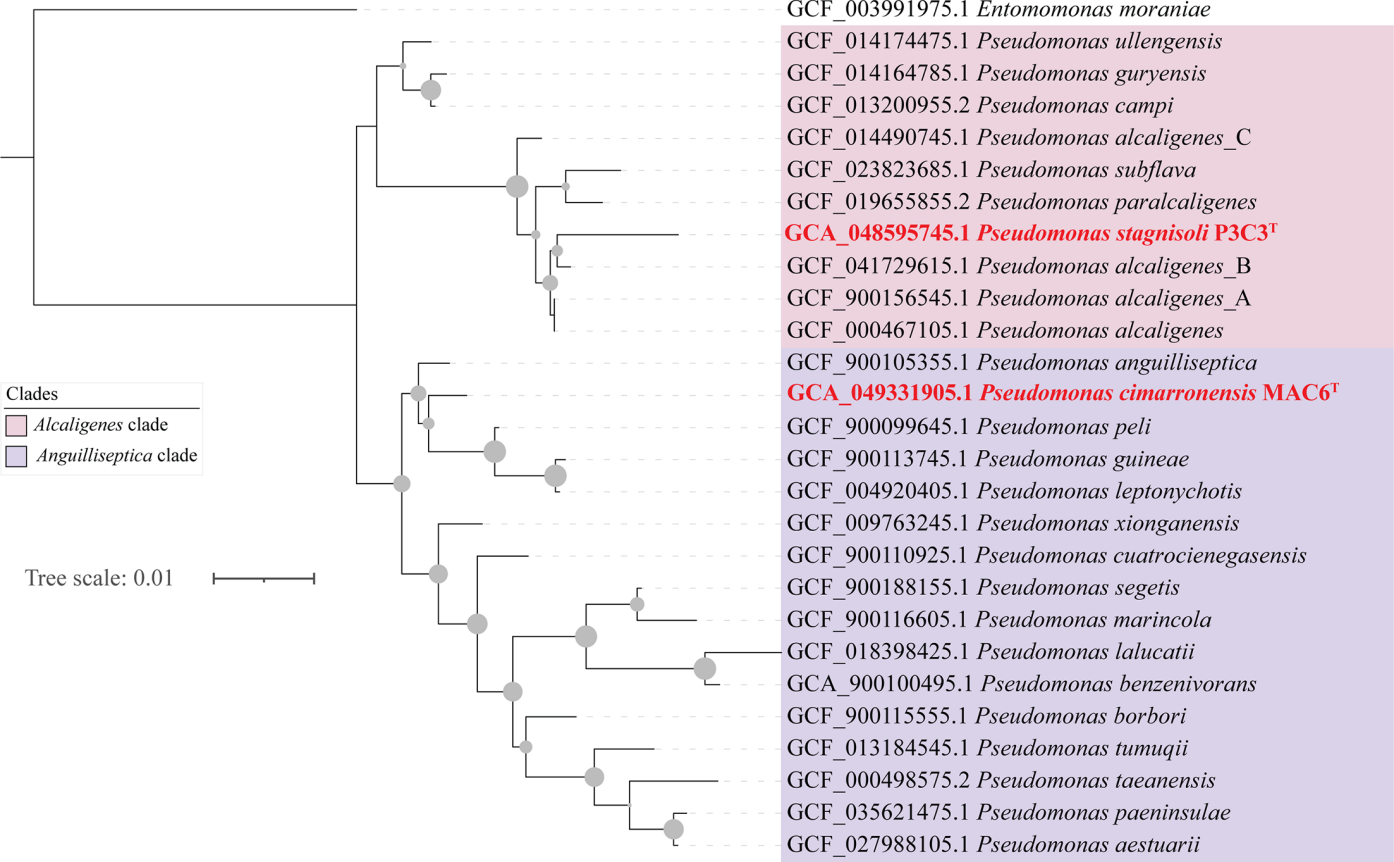
Phylogenetic tree based on 16S rRNA gene sequence showing the relationships with close relatives within *Pseudomonas* Alacaligenes and Anguilliseptica clades for P3C3^T^ and MAC6^T^. Bootstrap support values based on 100 replicates are shown as grey spheres for nodes with >70% support. Clades are colour-coded as shown in the legend. *E. moraniae* QZS01^T^ (MG768960) was used as the root of the tree. Bar, 0.01 substitutions per site.

A phylogenetic analysis using 16S rRNA gene sequences indicated that P3C3^T^ clustered in a distinct lineage with its closest validly published relatives being *Pseudomonas alcaligenes* (98.3%), *Pseudomonas paralcaligenes* (97.9%) and *Pseudomonas subflava* (97.8%) (Fig. 1). The monophyletic nature of the ‘Alcaligenes’ clade has previously been recognized (in [23]), and a recent phylogenomic study proposed accommodating members of this clade as a novel genus (*Aquipseudomonas*) [23]. On the other hand, strain MAC6^T^ clustered in a distinct lineage with its closest described relatives being *Pseudomonas anguilliseptica* (98.6%), *Pseudomonas peli* (98.6%), *Pseudomonas guineae* (97.7%), *Pseudomonas xionganensis* (98.1%), *Pseudomonas cuatrocienegasensis* (97.9%) and *Pseudomonas leptonychotis* (97.8%). (Fig. 1). This clade was labelled ‘Anguilliseptica’ clade in the same study [23] but was proposed to be retained as part of the genus *Pseudomonas* given its poor resolution and weak statistical support for its monophyletic nature in the conducted analysis.

## Whole-genome sequencing and comparative genomic analyses

To further clarify the taxonomic status of strains P3C3^T^ and MAC6^T^, their genomes were sequenced. Cell biomass was harvested from an overnight culture and stored in DNA/RNA Shield (Zymo, Irvine, CA). Subsequent DNA extraction, sequencing using an Oxford nanopore platform, gene calling and annotation were conducted using the services of a commercial provider (Plasmidsaurus, Eugene, OR) as previously described [24]. Assembly completeness and contamination were assessed using CheckM v1.2.2 [25]. Genome annotation was performed using the NCBI Prokaryotic Genome Annotation Pipeline v6.7 [26].

 To delineate species boundaries, overall genomic relatedness indices (OGRIs) between P3C3^T^ (GCA_048595745.1), MAC6^T^ (GCA_049331905.1) and the genome sequences of their close relatives were assessed according to the most recent proposed minimal standards for the use of genome data for the taxonomy of prokaryotes [27] [1,2]. Digital DNA–DNA hybridization (dDDH) values were calculated by uploading genome sequences to the Type Strain Genome Server (TYGS; https://tygs.dsmz.de) [28]. The average nucleotide identity (ANI) was calculated using EzBioCloud’s ANI Calculator, which employs the OrthoANIu algorithm [29]. The genomes of close relatives, representing the ‘Alacaligenes’ clade (*n*=9) and the ‘Anguilliseptica’ clade (*n*=15), in addition to those for P3C3^T^ and MAC6^T^, were also used for comparative taxonomic analysis, whereby phylogenomic trees were constructed using a concatenated alignment of 120 single-copy bacterial genes with Genome Taxonomy Database (GTDB) Toolkit [30]. A maximum-likelihood phylogenomic tree was constructed in RAxML using the PROTGAMMABLOSUM62 model and default parameters [31] as previously described [32].

The genome of strain P3C3^T^ comprises a single chromosome of 4,327,130 bp, with a G+C content of 64.5 mol%. The genome of P3C3^T^ contains a total of 4,122 genes, of which 3,978 are protein-coding genes, 61 are tRNA genes and 12 are rRNA genes (Table 1). In comparison, the MAC6^T^ genome consists of a single chromosome with 4,214,341 bp, with a G+C content of 60.5 mol%. The genome contains 3,955 total genes, including 3,827 protein-coding genes, 64 tRNA genes and 15 rRNA genes (Table 2).

**Table 1.** General genomic features of P3C3^T^ and its close relatives Strains: P3C3^T^; *P. alcaligenes* NBRC 14159^T^; *Pseudomonas tohonis* TUM 18999^T^; *Pseudomonas solani* Sm006^T^; *P. paralcaligenes* MRCP 1333^T^; *Pseudomonas otitidis* DSM 17224^T^.

Characteristic	P3C3^T^	*P. alcaligenes*	*P. tohonis*	*P. solani*	*P. paralcaligenes*	*P. otitidis*
**Genome accession**	GCA_048595745.1	GCA_000467105.1	GCA_012767755.2	GCA_026072635.1	GCA_019655855.2	GCA_900111835.1
**Genome size (Mb)**	4.3	4.8	6.8	6.7	5.4	6.3
**Number of contigs**	1	122	1	1	50	54
**G+C content (mol%)**	64.5	65	66.5	66.5	66.5	67
**Total genes**	4,122	4,614	6,222	6,072	5,064	5,937
**Protein-coding genes**	3,978	4,480	6,093	5,779	4,917	5,802
**tRNA genes**	61	54	64	63	53	55
**rRNA genes**	12	4	12	12	3	6

**Table 2.** General genomic features of MAC6^T^ and its close relatives Strains: MAC6^T^; *P. anguilliseptica* DSM 12111^T^; *P. peli* DSM 17833^T^; *Pseudomonas campi* S1-A32-2^T^; *P. xionganensis* R-22-3w-18^T^; *Pseudomonas guryensis* SR9^T^.

Characteristic	MAC6^T^	*P. anguilliseptica*	*P. peli*	*P. campi*	*P. xionganensis*	*P. guryensis*
**Genome accession**	GCA_049331905.1	GCA_900105355.1	GCA_900099645.1	GCA_013200955.2	GCA_009763245.1	GCA_014164785.1
**Genome size (Mb)**	4.2	5.2	4.5	4.4	4	4.3
**Number of contigs**	1	3	20	1	20	8
**G+C content (mol%)**	60.5	60	59.5	63.5	63	63.5
**Total genes**	3,955	5,205	4,345	4,089	3,739	4,007
**Protein-coding genes**	3,827	4,754	4,226	3,992	3,643	3,931
**tRNA genes**	64	64	54	58	52	54
**rRNA genes**	15	12	4	9	3	4

OGRIs for species delineation revealed ANI and dDDH values below the proposed species-level delineation thresholds (<95.0% and <70.0%, respectively) between P3C3^T^ and its closest relatives (members of the ‘Alcaligenes’ clade). Within validly published species, the highest ORGI values were observed between strain P3C3^T^ and *P. alcaligenes* (94.4 and 57% for ANI and dDDH, respectively) (Table 3). Despite the relatively high similarity, these values remain below the accepted thresholds for species delineation (as outlined by Riesco and Truhilo [27], supporting its distinction from *P. alcaligenes*). Interestingly, strain P3C3^T^ showed high ANI values to genomes of two isolates that have not been validly described: GCF_900156545.1 (97.0%) and GCF_900156135.1 (95.3%). Both genomes represent isolates obtained as part of a culture-based survey of the microbiome associated with the aquatic duckweed plant. The genomes are assigned to *P. alcaligenes_A* in the GTDB, in accordance with the GTDB practice of appointing an alphabetic suffix to genomes with relatedness to a described species. As such, strain P3C3^T^ (proposed name *P. stagnisoli*) is the appropriate representative of the unofficially designated *P. alcaligenes_A* in the GTDB naming hierarchy.

**Table 3.** Pairwise comparison of 16S rRNA gene similarity and OGRIs between P3C3^T^ (top panel), MAC6^T^ (bottom panel) and their close relatives Strains: (top panel) P3C3^T^; *P. alcaligenes* NBRC 14159^T^; *P. tohonis* TUM 18999^T^; *P. solani* Sm006^T^; *P. paralcaligenes* MRCP 1333^T^; *P. otitidis* DSM 17224^T^. (Bottom panel) MAC6^T^; *P. anguilliseptica* DSM 12111^T^; *P. peli* DSM 17833^T^; *P. campi* S1-A32-2^T^; *P. xionganensis* R-22-3 w-18^T^; *P. guryensis* SR9^T^.

Strain	16S rRNA gene accession	16S rRNA gene similarity (%)	Genome accession	ANI (%)	dDDH (%)
**P3C3ᵀ**	PV166458	100.0	GCA_048595745.1	100.0	100.0
***P. alcaligenes***	BATI01000076	98.3	GCA_000467105.1	94.4	57
	LC645211	97.4	GCA_012767755.2	81.1	24.2
***P. solani***	LC744517	97.4	GCA_026072635.1	80.7	23.6
***P. paralcaligenes***	MT604974	97.1	GCA_019655855.2	85.3	31.8
***P. otitidis***	MF574358	96.8	GCA_900111835.1	80.3	23.5
	**16S rRNA gene accession**	**16S rRNA gene similarity (%)**	**Genome accession**	**ANI (%)**	**dDDH (%)**
**MAC6ᵀ**	PV166459	100.0	GCA_049331905.1	100.0	100.0
***P. anguilliseptica***	FNSC01000001	98.6	GCA_900105355.1	88.4	35.7
***P. peli***	AM114534	98.6	GCA_900099645.1	87.7	34.3
***P. campi***	MT415401	98.1	GCA_013200955.2	80.1	23.5
***P. xionganensis***	MN593501	98.1	GCA_009763245.1	82.2	25.3
***P. guryensis***	MN658350	98.1	GCA_014164785.1	79.6	22.9

Similarly, OGRIs for strain MCA6^T^ and its closest relatives were lower than the proposed thresholds for species-level delineation. The highest similarity was between strain MAC6^T^ and *P. anguilliseptica* (88.4 and 35.7% for ANI and dDDH, respectively) (Table 3).

**Fig. 2.**
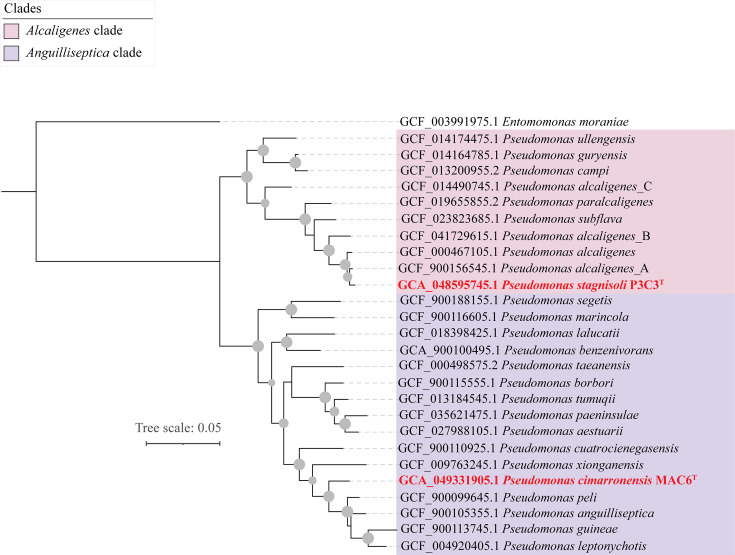
Core-genome phylogenomic tree using the GTDB concatenated alignment of 120 single-copy marker gene sequences showing the relationships with close relatives within *Pseudomonas* Alcaligenes and Anguilliseptica clades for P3C3^T^ and MAC6^T^. Bootstrap support values are shown as grey spheres for nodes with >70% support. Clades are colour-coded as shown in the legend. The tree was rooted using *E. moraniae* QZS01^T^ (GCF_003991975.1). Bar, 0.1 substitutions per site.

Phylogenomic analysis confirmed the affiliation of strains P3C3^T^ and MAC6^T^ with the genus *Pseudomonas*, as well as their distinction from all other described *Pseudomonas* species (Fig. 2). Both strains clustered with species assigned to the broader *Pseudomonas*_E clade in the GTDB taxonomic outline [33]. Similar to the 16S rRNA gene analysis and recent phylogenomic efforts [23], the closest relatives of P3C3^T^ were members of the ‘Alcaligenes’ clade, while the closest relatives of MAC6^T^ were members of the ‘Anguilliseptica’ clade (Fig. 2).

Collectively, the results of comparative OGRI indices and phylogenomic analysis strongly indicate that both strains represent two novel, distinct species within the genus *Pseudomonas*.

## Metabolic reconstruction and functional genomic analysis

To reconstruct the metabolic processes predicted in both genomes, we used Ghost_Koala to assign KEGG orthology numbers to the predicted proteins in strains P3C3^T^ and MAC6^T^. In addition, we assigned KEGG orthology numbers to the closely related genomes in the ‘Alcaligenes’ and ‘Anguilliseptica’ clades. KEGG Mapper was then used to predict the metabolic capabilities encoded in these genomes. Strain P3C3^T^ was compared to the genomes of *P. alcaligenes*_A (GCF_900156545.1), *P. alcaligenes* (GCF_000467105.1), *P. alcaligenes*_B (GCF_003205495.1), *P. alcaligenes*_C (GCF_014490745.1), *P. paralcaligenes* (GCF_019655855.2) and *P. subflava* (GCF_023823685.1), while strain MAC6^T^ was compared to the genomes of *P. leptonychotis* (GCF_004920405.1), *P. xionganensis* (GCF_009763245.1), *P. peli* (GCF_900099645.1), *P. anguilliseptica* (GCF_900105355.1), *P. cuatrocienegasensis* (GCF_900110925.1) and *P. guineae* (GCF_900113745.1).

Analysis of the P3C3^T^ and the MAC6^T^ genomes revealed characteristics typical of members of the genus *Pseudomonas* (Tables S1 and S2, available in the online Supplementary Material). This included an aerobic heterotrophic lifestyle (EMP pathway, pyruvate dehydrogenase complex for pyruvate oxidative decarboxylation, a complete TCA cycle with α-ketoglutarate dehydrogenase and a complete electron transport system with NADH-quinone oxidoreductase [EC:7.1.1.2], succinate dehydrogenase [EC:1.3.5.1], cytochrome bc1 complex [EC:7.1.1.8] and two different cytochrome oxidases [EC:7.1.1.9] with different oxygen affinities; the low oxygen affinity aa3-type and the high oxygen affinity cbb3-type, in addition to the F-type ATPase for oxidative phosphorylation). Capacities for substrate-level phosphorylation using the phosphate acetyltransferase-acetate kinase pathway and acetyl-CoA synthetase [EC:6.2.1.1] are encoded in both genomes, while P3C3^T^ additionally encodes succinyl-CoA:acetate CoA-transferase [EC:2.8.3.18] for acetyl-CoA conversion to acetate. The genomes also encode glyoxylate shunt enzymes (isocitrate lyase and malate synthase) and the 2-methylcitrate cycle enzymes, which enable the organism to utilize organic acids such as acetate and propionate, respectively. P3C3^T^ genome encoded the capacity for anaerobic respiration using nitrate (through dissimilatory nitrate reduction to ammonium). Both strains encoded the capacity for glycine, proline, histidine and tyrosine degradation, with P3C3^T^ additionally encoding lysine and hydroxylproline degradation, and MAC6^T^ additionally encoding leucine and betaine degradation. A limited CAZyome was identified in the genomes, with capacities to degrade alginate encoded only in the P3C3^T^ genome. Finally, P3C3^T^ encoded the capacity for aerobic alkane degradation to fatty acids via alkane 1-monooxygenase [EC:1.14.15.3], alcohol dehydrogenase and aldehyde dehydrogenase.

Both strains are predicted to be motile, as they encode a complete flagellar and twitching motility (via type IV pili) machinery. Both are also predicted to be chemotactic, based on the presence of a complete bacterial chemotaxis pathway. Secretion systems encoded in both genomes included the general secretory pathway (type II and the associated Sec pathway) and the Tat pathway.

Predictably, predicted metabolic features from the genomes of strains P3C3^T^ and MAC6^T^ were highly similar to those from the genomes of their closest relatives. Nevertheless, a few differences were identified. For example, the capacity to degrade benzoate, express fimbriae, synthesize type I, III, IV and VI secretion systems and utilize starch/glycogen was identified in some members of the ‘Alcaligenes’ and/or ‘Anguilliseptica’ clades, but not P3C3^T^ and MAC6^T^. Details of such differences are provided in Tables S1 and S2.

## Ecological distribution patterns

We used two distinct approaches, mOTUs [Marker Gene (MG)-based operational taxonomic units] and Sandpiper, to gauge the ecological distribution pattern of both strains on a global scale. The mOTUs database [34] [comprised of reference genomes clustered into 11,915 ref-mOTUs and a collection of both metagenomic contigs, MAGs and SAGs (*n*=21,655)] was searched using mOTUs extender [34] for other genome or MAG representatives of the two species. Five representatives of *P. stagnisoli* were available in the mOTUs database. These included two genomes in ref_mOTU_v3_00227 of isolates from duckweed microbiome, referred to as *P. alcaligenes*_A in the GTDB database mentioned above; two genomes in ref_mOTU_v3_00226, one obtained from Apalachicola Bay oysters’ mantle and the other obtained from a human blood infection; and a fifth MAG that was assembled and binned from a wastewater sample.

In addition, we queried Sandpiper [35], an interface that relies on a recently developed tool (SingleM) for mapping of metagenomic reads to genomes, to determine the occurrence and relative abundance of P3C3^T^ in 248,559 metagenomic datasets. Results showed that representatives of P3C3^T^ were identified in 1,553 datasets, the majority of which (*n*=1,042) were from engineered environments, followed by 214 from freshwater environments and 123 from host-associated environments. This pattern is broadly similar to the environments from which genomic assemblies were obtained (four from host-associated environments, one from a wastewater treatment sample and one, described here, from a freshwater environment) in the mOTUs-based analysis, and both analyses hence attest to the capacity of P3C3^T^ to inhabit a wide range of disparate environmental biomes.

On the other hand, no MAC6^T^ representative was identified in the mOTUs database. Also, since Sandpiper [35] provides only pre-calculated analyses for all species representatives that are currently recognized in the GTDB R226 release, such an analysis was not possible for MAC6^T^. We hope that, with the next GTDB update, a similar analysis using Sandpiper for MAC6^T^ will be possible.

## Morphology and physiology

The Gram stain reaction was determined using the Gram Stain Kit (Becton Dickinson, Franklin Lakes, NJ) according to the manufacturer’s instructions. The morphology and size of P3C3^T^ (Fig. S1) and MAC6^T^ (Fig. S2) were measured by scanning electron microscopy (Thermo Fisher, FEI, Quanta 600F). Aerotolerance was determined by inoculating P3C3^T^ and MAC6^T^ onto TSA and incubating them for 1 week at an optimal temperature inside an anaerobic chamber (Coy Laboratory Products, Grass Lake, MI) containing 90% nitrogen and 10% hydrogen. Temperature range tolerance for growth was determined in tryptic soy broth (TSB) at 4 °C, 20 °C, 25 °C, 37 °C, 42 °C, 45 °C and 50 °C. Salt tolerance was determined in TSB and tested at 0.5% (w/v) NaCl and between 1.0% (w/v) and 10.0% (w/v), in increments of 1.0% (w/v); pH ranges for growth were determined between 3.0 and 10.0, in increments of 1.0, and all tests were performed in triplicate. For pH experiments, Good’s buffers were prepared as previously described [36]. Optimal growth conditions were determined by OD (OD600) using a Multiskan Go Microplate Spectrophotometer (Thermo Scientific, Waltham, MA). An increase in OD600 greater than 0.1 after 5 days of incubation was considered growth. The oxidase test was performed using commercial testing strips (Hardy Diagnostics, Santa Maria, CA), and the catalase test was conducted by adding a drop of 1% hydrogen peroxide to the biomass on a glass slide. Additional physiological and biochemical characteristics, such as carbon substrate utilization and antimicrobial susceptibility, were determined using the GENIII microplate system (BIOLOG, Hayward, CA) and the API ZYM kit (bioMérieux, Durham, NC), according to the manufacturers' protocols.

Strains P3C3^T^ and MAC6^T^ were identified as rod-shaped, Gram-stain-negative and strictly aerobic bacteria. Dimensionally, P3C3^T^ was 2.2±0.4 µm long and 0.4±0.1 µm wide, while MAC6^T^ averaged 2.3±0.3 µm long and 0.5±0.1 µm in width (*n*=10 for both strains, values represent mean±sd). After 48 h of incubation at 35 °C on TSA, P3C3^T^ colonies were circular, rough and pale yellow, whereas the colonies of MAC6^T^ on TSA were circular, smooth and pale yellow after 48 h at 30 °C. Strain P3C3^T^ grew within a temperature range of 25–40 °C, with optimal growth at 35 °C; within a salt range of 0.5 and 3.0% (v/w) NaCl, with optimal growth at 0.5% (w/v) NaCl; and within a pH range of 5.0–7.0, with optimum growth at a pH of 6.0. Strain MAC6^T^ grew within a temperature range of 4 and 40 °C, with optimal growth at 30 °C; within a salt range of 0.5 and 5.0% (v/w) NaCl, with optimal growth at 4.0% (w/v) NaCl; and within a pH range of 6.0–9.0, with optimal growth at a pH of 6.0.

Detailed phenotypic characteristics of P3C3^T^ and MAC6^T^ are provided in their respective species descriptions. Distinguishing phenotypic characteristics between strains P3C3^T^ and MAC6^T^ and close relatives are listed in Tables 4 and 5. Both strains were capable of utilizing l-arginine, l-aspartic acid, l-glutamic acid, methyl pyruvate, l-lactic acid, citric acid, l-malic acid, potassium tellurite, Tween-40, α-ketobutyric acid and glucose as sole carbon sources. Additionally, resistance to lincomycin, vancomycin and guanidine HCl was observed for both P3C3^T^ and MAC6^T^. Notably, P3C3^T^ and its closest neighbours differ in lacking phosphatase and leucine arylamidase activities (Table 4). Additionally, MAC6^T^ and its closest relatives differ in that lipase (C14) is absent (Table 5). A complete list of the Biolog GEN III microplate system results for P3C3^T^ and MAC6^T^ is given in Table S3.

**Table 4.** Key physiological parameters and enzymatic activities distinguishing P3C3^T^ and its close relatives Strains: P3C3^T^ (data from this study); *P. alcaligenes* NBRC 14159^T*^; *P. tohonis* TUM 18999^T^ [42]; *P. solani* Sm006^T^ [43]; *P. paralcaligenes* MRCP 1333^T^ [44]; *P. otitidis* DSM 17224^T^*.

Characteristic	P3C3^T^	*P*. *alcaligenes*	*P*. *tohonis*	*P*. *solani*	*P*. *paralcaligenes*	*P*. *otitidis*
**Physiological parameters**						
**Temperature range (°C)**	25–40	10–41	20–42	12–37	15–40	7–45
**Optimum temperature (°C)**	35	NR	30–35	NR	25–35	NR
**pH range**	5.0–7.0	NR	5.5–9.5	6.0–8.0	6.0–9.5	5.0–10.0
**Optimum pH**	6.0	NR	5.5–8.5	NR	7.0–8.0	NR
**Salinity range (%)**	0.5–3.0	0.0–2.0	1.0–5.0	0.0–5.0	1.0–3.0	1.0–4.0
**Optimum salinity (%)**	0.5	NR	1.0–4.0	NR	1.0–2.0	NR
**API ZYM**						
**Alkaline phosphatase**	−	+	NR	+	**+**	+
**Lipase (C14)**	−	+	NR	−	**+**	+
**Leucine arylamidase**	−	+	NR	+	**+**	+
**Trypsin**	−	+	NR	+	NR	−

*API ZYM data for *P. alcaligenes* and *P. otitidis* have been taken from https://bacdive.dsmz.de/. The following characteristics were shared between all isolates, where data were reported: esterase (C4) (+), esterase lipase (C8) (+), valine arylamidase (−), cystine arylamidase (−), α-chymotrypsin (−), acid phosphatase (+), naphthol-AS-BI-phosphohydrolase (+), α-galactosidase (−), β-galactosidase (−), β-glucuronidase (−), α-glucosidase (−), β-glucosidase (−), N-acetyl-β-glucosaminidase (−), α-mannosidase (−) and α-fucosidase (−); NR, not reported.

**Table 5.** Physiological parameters and enzymatic activity of MAC6^T^ and its close relatives Strains: MAC6^T^ (data from this study); *P. anguilliseptica* DSM 12111^T*^; *P. peli* DSM 17833^T^ [40]; *P. campi* S1-A32-2^T^ [45]; *P. xionganensis* R-22-3 w-18^T^ [46]; *P. guryensis* SR9^T^ [42].

Characteristic	MAC6^T^	*P. anguilliseptica*	*P. peli*	*P. campi*	*P. xionganensis*	*P. guryensis*
**Physiological parameters**						
**Temperature range (°C)**	4–40	10–30	28–37	25–37	15–37	10–37
**Optimum temperature (°C)**	30	NR	NR	25	30	30
**pH range**	6.0–9.0	NR	NR	6.0–9.0	7.0–10.0	4.0–10.0
**Optimum pH**	6.0	NR	NR	NR	8.0	8.0
**Salinity range (%)**	0.5–5.0	0.0–2.0	NR	0.0–2.0	NR	0.0–1.0
**Optimum salinity (%)**	4.0	NR	NR	NR	NR	0.0
**API ZYM**						
**Alkaline phosphatase**	+	+	−	−	+	−
**Esterase (C4)**	+	+	−	+	+	+
**Lipase (C14)**	−	+	+	+	+	+
**Leucine arylamidase**	+	−	NR	+	+	+
**Valine arylamidase**	−	−	−	+	+	+
**Cystine arylamidase**	−	−	NR	−	+	−
**Trypsin**	−	−	−	−	+	+
**α-Chymotrypsin**	−	−	−	−	+	−
**Acid phosphatase**	**+**	+	−	−	−	+
**Naphthol-AS-BI-phosphohydrolase**	**+**	−	+	+	+	+

*API ZYM data for *P. anguilliseptica* have been taken from https://bacdive.dsmz.de/. The following characteristics were shared between all isolates, where data were reported: α-galactosidase (−), β-galactosidase (−), β-glucuronidase (−), α-glucosidase (−), β-glucosidase (−), N-acetyl-β-glucosaminidase (−), α-mannosidase (−), α-fucosidase (−) and esterase lipase (C8) (+); NR, not reported.

## Taxonomic conclusions

Based on their phylogenomic, physiological and genotypic features, strains P3C3^T^ and MAC6^T^ are proposed to represent two novel species of the genus *Pseudomonas*. The genus *Pseudomonas* has long been recognized as paraphyletic, with various studies grouping its members into multiple clades lacking a common evolutionary ancestor, and additional genera (*Azomonas*, *Azotobacter* and *Chryseomonas*) clustering between these clades [23,37,41]. Multiple recent efforts have been conducted to resolve the taxonomy of the genus by proposing new genera for various clades (e.g. *Atopomonas*, *Halopseudomonas* and *Stutzerimonas*) [37,41] and assigning various species to existing genera. 16S rRNA gene-based analysis (Fig. 1) and whole-genome phylogenomic analysis (Fig. 2) assigned strain P3C3^T^ to the ‘Alcaligenes’ clade. A proposal to accommodate members of this clade into a new genus (*Aquipseudomonas* gen. nov.) has recently been put forth [37]. However, such a proposal does not mean that the genus name *Pseudomonas* can no longer be used to describe novel species in this clade, as it remains validly published and has not been placed on the list of *nomina rejicienda* by the Judicial Commission of the ICSP. Further, values reported for the Average Amino Acid Identity (AAI) and Percentage of Conserved Proteins (POCP) between members of this clade and other taxa within the genus *Pseudomonas sensu lato* ranged between 0.68–0.79 (AAI) and 0.49–0.75 (POCP) [23]. Such values are higher (i.e. exhibit greater similarity) than the proposed values for genus-level designation in the most recent minimal standards for the use of genome data in the taxonomy of prokaryotes [27]. Therefore, we opt to retain the designation of strain P3C3^T^ as a novel species in the genus *Pseudomonas* in this study, for which the name *P. stagnisoli* (=P3 C3^T^=CCM 9468^T^=CECT 31230^T^=DSM 119661^T^) sp. nov. is proposed.

Regarding strain MAC6^T^, its assignment to the genus *Pseudomonas* is supported by 16S rRNA and whole-genome phylogenomic analyses. Strain MAC6^T^ clustered with a clade of 12 species, referred to as the ‘Anguilliseptica’ clade in both analyses (Figs. 1 and 2). However, while this clade is distinct from the clade defined by the type species *Pseudomonas aeruginosa*, weak statistical support to confirm its monophyletic nature was obtained, and hence, the designation of all species in this clade to the genus *Pseudomonas* was retained. Therefore, we assign strain MAC6^T^ as a novel species in the genus *Pseudomonas* in this study, for which the name *P. cimarronensis* (=MAC6^T^=CCM 9470^T^=CCUG 78313^T^=CECT 31231^T^) sp. nov. is proposed.

## Description of *Pseudomonas stagnisoli* sp. nov.

*Pseudomonas stagnisoli* (stag.ni.so’li. L. neut. n. *stagnum*, pond; L. neut. n. *solum*, soil; N.L. gen. n. *stagnisoli*, of pond soil).

Cells are Gram-stain-negative, non-spore-forming, obligately aerobic, rod-shaped, motile bacteria. The average length is 2.2±0.4 µm long, and the average width is 0.4±0.1 µm (*n*=10, mean±sd). Colonies on TSA are rough, circular and pale yellow after 48 h of incubation at 35 °C. Growth occurs at temperatures between 25 and 40 °C (optimum 35 °C), between NaCl 0.5 and 3.0% (v/w) [optimum 0.5% (w/v)] and between pH 5.0 and 7.0 (optimum 6.0). Cells utilized l-arginine, l-aspartic acid, l-glutamic acid, methyl pyruvate, l-lactic acid, citric acid, l-malic acid, Tween-40, γ-aminobutyric acid, α-ketobutyric acid, propionic acid and acetic acid for growth. Cells grew in the presence of rifamycin SV, lincomycin, vancomycin, 1% sodium lactate, guanidine HCl, Niaproof 4, tetrazolium violet, tetrazolium blue and potassium tellurite. Cells were catalase and oxidase positive. Using the API ZYM test system, positive reactions are observed for esterase (C4), esterase lipase (C8), acid phosphatase and naphthol-AS-BI-phosphohydrolase.

The type strain P3C3^T^ (=CCM 9468^T^=CECT 31230^T^=DSM 119661^T^) was isolated from a sediment taken from the Innovation Way Drive Pond, Stillwater, OK. The DNA G+C content of the strain is 64.5 mol%. The GenBank accession numbers of the whole genome and 16S rRNA gene are GCA_048595745.1 and PV166458, respectively.

## Description of *Pseudomonas cimarronensis* sp. nov.

*Pseudomonas cimarronensis* (ci.mar.ron.en’sis. N.L. fem. adj. *cimarronensis*, pertaining to the Cimarron River).

Cells are Gram-stain-negative, non-spore-forming, obligately aerobic, rod-shaped, motile bacteria. The average length is 2.3±0.3 µm long, and the average width is 0.5±0.1 µm (*n*=10, mean±sd). Colonies on TSA are smooth, circular and pale yellow after 48 h of incubation at 30 °C. Growth occurs at temperatures between 4 and 40 °C (optimum 30 °C), between NaCl 0.5 and 5.0% (v/w) [optimum 4.0% (w/v)] and between pH 6.0 and 9.0 (optimum 6.0). Cells utilized d-galactose, 3-methyl glucose, d-fucose, l-fucose, l-rhamnose, d-fructose-6-PO4, l-alanine, l-arginine, l-aspartic acid, l-glutamic acid, l-histidine, l-galactonic acid lactone, methyl pyruvate, l-lactic acid, citric acid, l-malic acid, Tween-40, α-ketobutyric acid and acetoacetic acid for growth. Cells grew in the presence of lincomycin, vancomycin, 1% sodium lactate, guanidine HCl, Niaproof 4 and potassium tellurite. Cells were catalase and oxidase positive. Using the API ZYM test system, positive reactions are observed for alkaline phosphatase, esterase (C4), esterase lipase (C8), leucine arylamidase, acid phosphatase and naphthol-AS-BI-phosphohydrolase.

The type strain MAC6^T^ (=CCM 9470^T^=CCUG 78313^T^=CECT 31231^T^) was isolated from a sediment sample collected from the Cimarron River, Perkins, OK. The DNA G+C content of the strain is 60.5 mol%. The GenBank accession numbers of the whole genome and 16S rRNA gene are GCA_049331905.1 and PV166459, respectively.

## Supplementary material

10.1099/ijsem.0.007075 Uncited Supplementary Material 1.

10.1099/ijsem.0.007075 Uncited Supplementary Material 2.

## Abbreviations

AAI: Average Amino Acid Identity
ANI: average nucleotide identity
CCM: Czech Collection of Microorganisms
CCUG: Culture Collection University of Gothenburg
CECT: Spanish Type Culture Collection
dDDH: digital DNA–DNA hybridization
DSMZ: German Collection of Microorganisms and Cell Cultures
GTDB: Genome Taxonomy Database
MAC: MacConkey Agar
mOTUs: Marker Gene (MG)-based operational taxonomic units
OGRIs: overall genomic relatedness indices
POCP: Percentage of Conserved Proteins
R2A: Reasoner's 2A Agar
TSA: Tryptic Soy Agar
TSB: tryptic soy broth
TYGS: Type Strain Genome Server
