# Supplementary material for: Description of Pseudomonas stagnisoli sp. nov. and Pseudomonas cimarronensis sp. nov., isolated from freshwater sediments
Source: Int J Syst Evol Microbiol. 2026 Feb 16;76(2):007075. doi: 10.1099/ijsem.0.007075 (PMC12911915; doi:10.1099/ijsem.0.007075)

Supplemental Figure 1: Scanning electron micrograph of P3C3^T.

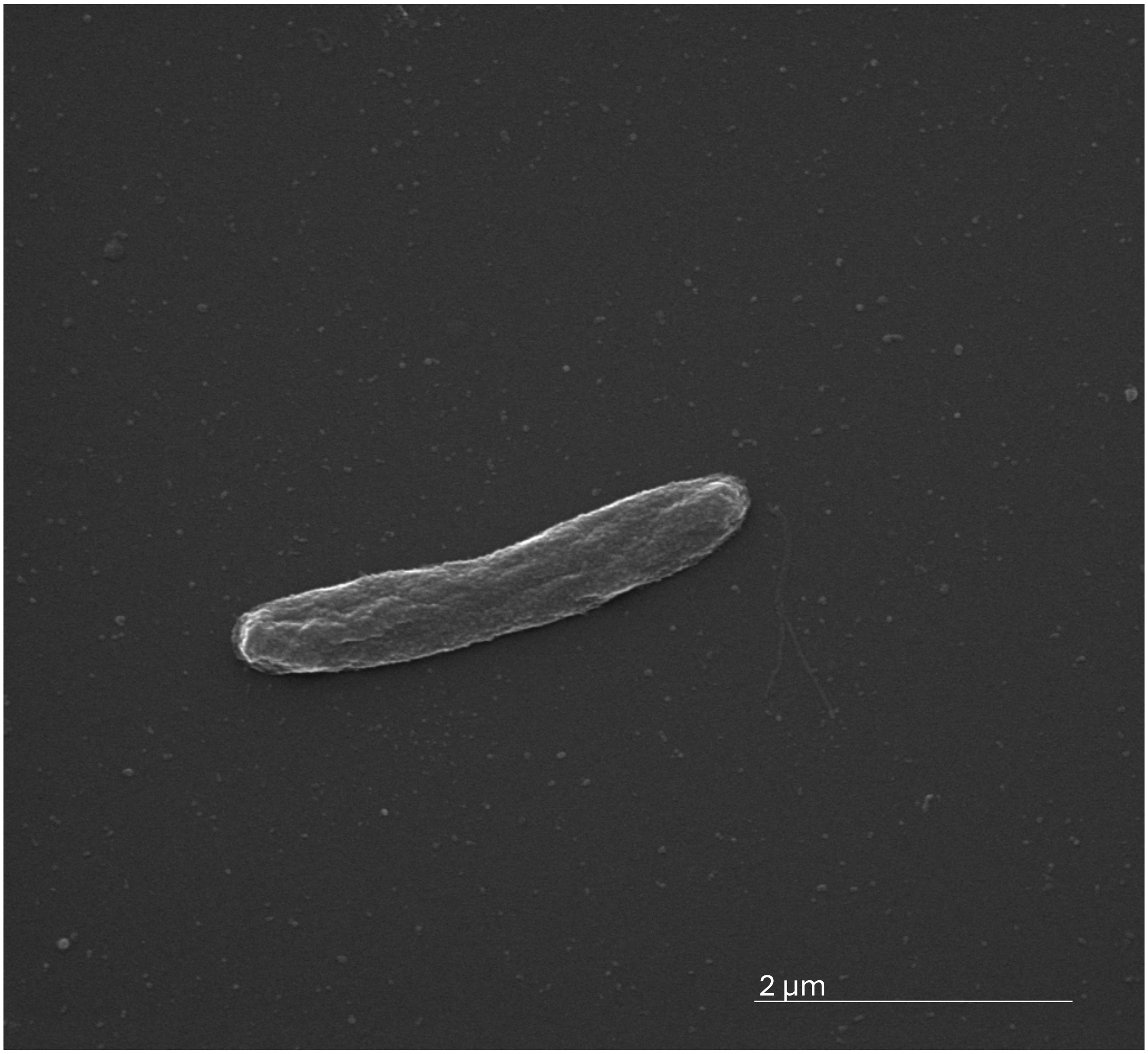

Supplemental Figure 2: Scanning electron micrograph of MAC6^T

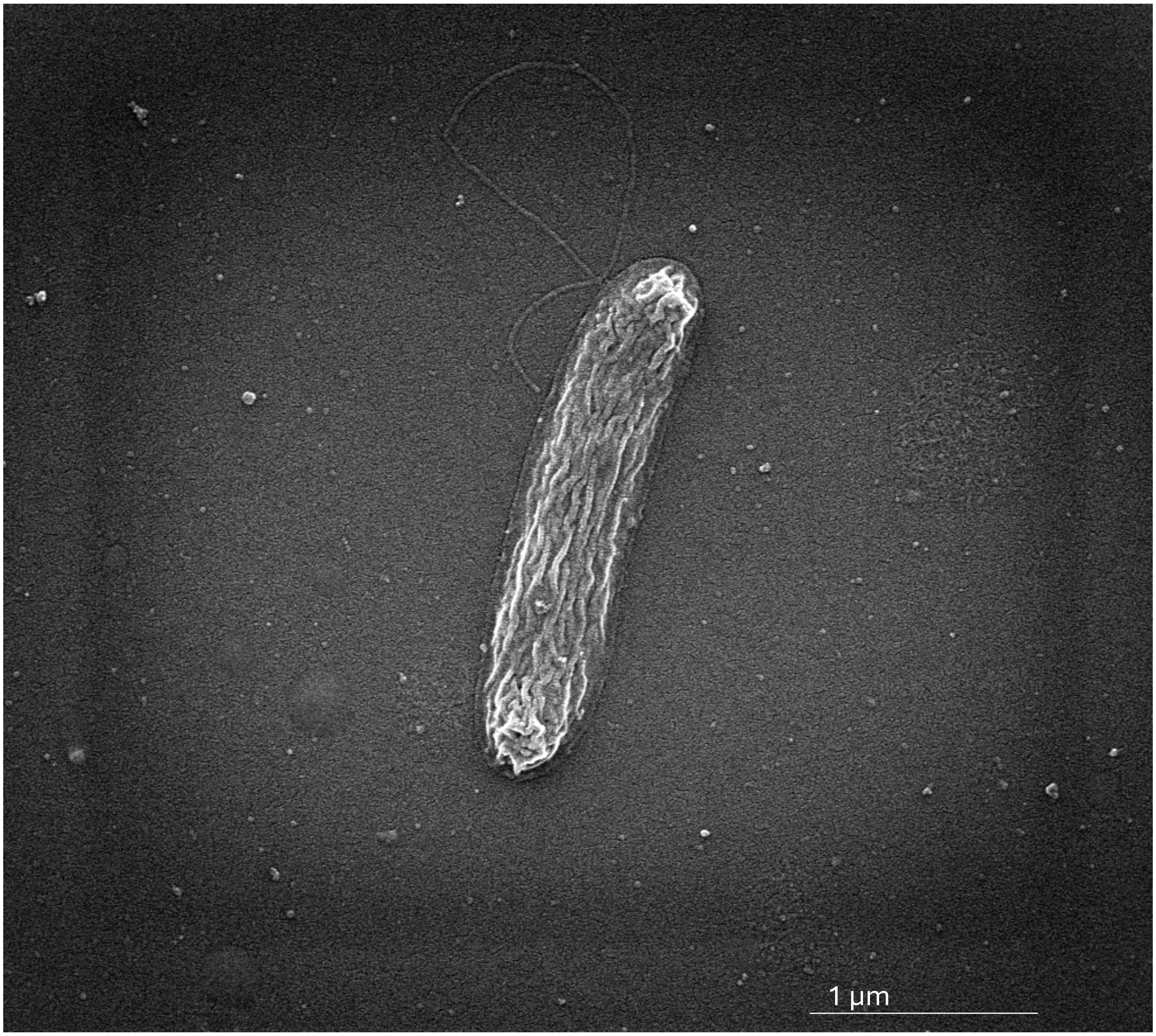

Supplement: Uncited Supplementary Material 1. [file ijsem-76-07075-s001.pdf]
